# A Common Medication With a Rare Side Effect: A Case Report of Statin-Induced Necrotizing Autoimmune Myopathy

**DOI:** 10.7759/cureus.83589

**Published:** 2025-05-06

**Authors:** Meghan Grossmann, Madison Drallmeier, Michelle Jin, Eric Scher

**Affiliations:** 1 Internal Medicine, Detroit Medical Center, Detroit, USA; 2 Internal Medicine, Henry Ford Health System, Detroit, USA; 3 Pathology, Henry Ford Health System, Detroit, USA

**Keywords:** adverse drug reaction, anti-hmg-coa reductase antibody, anti-signal recognition particle antibody, myopathy, statin-induced necrotizing autoimmune myopathy, statins

## Abstract

Statins are commonly used for their cholesterol-lowering properties and are prescribed for primary and secondary prevention of cardiovascular disease. Although they are generally well tolerated, some individuals may experience a rare and serious side effect in which autoantibodies develop and lead to a necrotizing myopathy. Here we present a case of a middle-aged woman chronically on atorvastatin for her hyperlipidemia who acutely developed myalgias, muscle weakness, and persistently elevated creatine kinase, who was eventually diagnosed with statin-induced necrotizing autoimmune myopathy.

## Introduction

Statins are one of the most widely prescribed medications in the United States. These agents affect the cholesterol synthesis pathway by inhibiting the enzyme hydroxymethylglutaryl-CoA (HMG-CoA) reductase and are useful in lowering the risk of cardiovascular events. Side effects of this drug class commonly include myalgias and weakness, which are benign and often resolve after discontinuation of the medication. However, a more serious complication is statin-induced necrotizing autoimmune myopathy (SINAM). This occurs in less than 0.01% of people on a statin medication and is characterized by progressive loss of proximal muscle strength, significant increase in creatinine kinase levels, and often the presence of autoantibodies [1]. SINAM, unlike the more benign myalgias, does not improve with cessation of the statin. It is imperative to diagnose and begin treatment early for improved outcomes. Treatment is focused on immunosuppression with the use of steroids, methotrexate, intravenous immune globulin (IVIG), rituximab, and/or azathioprine. Below, we describe a patient who was diagnosed with this rare adverse reaction to their statin. This article was previously presented as a poster at the 2025 ACP Internal Medicine Meeting on April 4, 2025.

## Case presentation

A 52-year-old female with a history of diabetes mellitus on metformin and semaglutide, as well as hyperlipidemia on atorvastatin, presented with 3 weeks of gradual weakness and myalgias. She initially presented to her primary care physician (PCP) and underwent testing, which noted an elevation in creatine kinase (CK), alanine aminotransferase (ALT), and aspartate aminotransferase (AST). Given her symptoms and laboratory results, her PCP discontinued her statin and recommended increasing her hydration. However, the patient continued to have myalgias and presented to an emergency department. At that time, she was noted to have up-trending CK; she was fluid resuscitated and discharged. The patient followed up with her PCP and reported persistent symptoms, and she was sent to a tertiary system for further investigation.

Upon presentation to a tertiary center, she was started on continuous fluids and admitted under internal medicine. Physical exam was remarkable for bilateral symmetrical proximal muscle weakness of the quadriceps and adductors. Initial workup revealed an elevated erythrocyte sedimentation rate (ESR), C-reactive protein (CRP), and continued elevation in CK and liver transaminases. She also had a positive antinuclear antibody (ANA) (Table 1).

**Table 1:** Initial Remarkable Labs at Presentation AST: aspartate aminotransferase; SGOT: serum glutamic oxaloacetic transaminase; ALT: alanine aminotransferase; SGPT: serum glutamic pyruvic transaminase; CK: creatine kinase; ESR: erythrocyte sedimentation rate; CRP: c-reactive protein; ANA: antinuclear antibody

	Result	Reference Range
AST/SGOT	1,124	<35 IU/L
ALT/SGPT	671	<52 IU/L
CK	33,374	<178 IU/L
ESR	43	<30 mm/hr
CRP	0.6	<0.5 mg/dL
ANA	>1:640; homogenous pattern	Negative, <1:80 titer

There was a concern for autoimmune-mediated myositis given the positive ANA. Additional testing was completed, including a myositis panel (testing for anti-Jo-1, anti-PL-7, anti- PL-12, anti-OJ, anti-MI-2, anti-TIF1 gamma, MDA-5, anti-NXP-2, anti-PM/Scl-100, anti-U3 ribonucleoprotein (RNP), anti-U2 RNP, anti-U1-RNP, anti-Ku, and anti-SS-A kD), rheumatoid factor, double stranded deoxyribonucleic acid (ds-DNA), anti-Ro/SSA, anti-La/SSB, and anti-signal recognition particle (SRP) that all returned negative. However, anti-hydroxymethylglutaryl-CoA reductase (anti-HMGCR) antibodies returned elevated (Table 2).

**Table 2:** Myositis Panel and Autoimmune Serologies ds-DNA: double stranded deoxyribonucleic acid; anti-SRP: anti-signal recognition particle; anti-HMGCR: anti-hydroxymethylglutaryl-CoA reductase; RNP: ribonucleoprotein

	Result	Reference Range
Anti-Jo-1	<20	<20 units
Anti-PL-7	Negative	Negative
Anti-PL-12	Negative	Negative
Anti-OJ	Negative	Negative
Anti-MI-2	Negative	Negative
Anti-TIF1 gamma	<20	<20 units
MDA-5	<20	<20 units
Anti-NXP-2	<20	<20 units
anti-PM/Scl-100	<20	<20 units
anti-U3 RNP	Negative	Negative
anti-U2 RNP	Negative	Negative
anti-U1-RNP	<20	<20 units
anti-Ku	Negative	Negative
anti-SS-A kD	<20	<20 units
Rheumatoid factor	<10	<10 units
ds-DNA	Negative	Negative
anti-Ro/SSA	<0.2	<0.2 units
anti-La/SSB	<0.2	<0.2 units
anti-SRP	Negative	Negative
anti-HMGCR	>200	0-19 units

**Figure 1:**
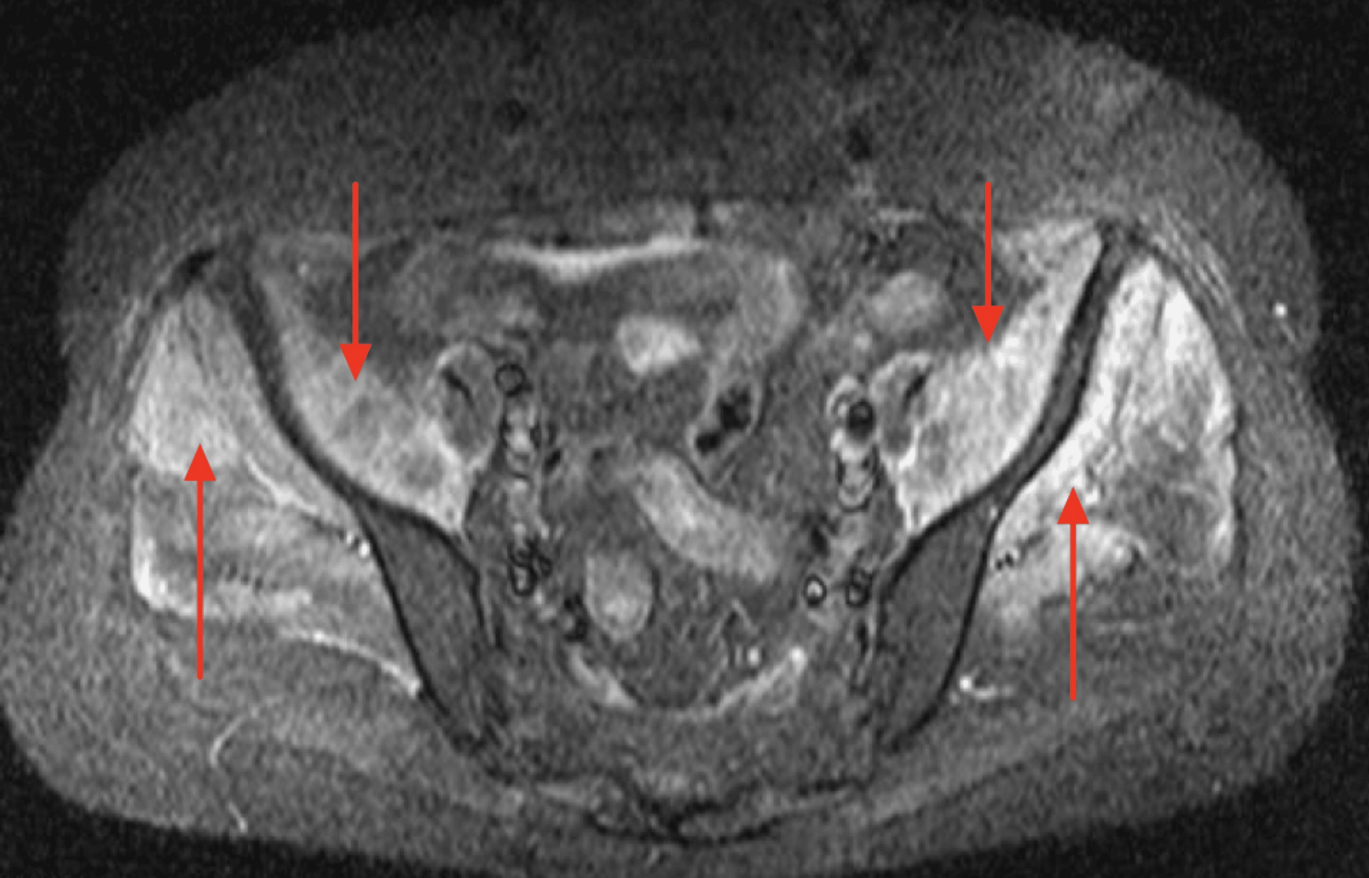
MRI of the Pelvic Muscles Revealing Edema Intense increased signaling in the iliacus, indicated by the red downward arrow, and gluteal muscles, indicated by the red upward pointing arrows, indicating edema within the muscles.

**Figure 2:**
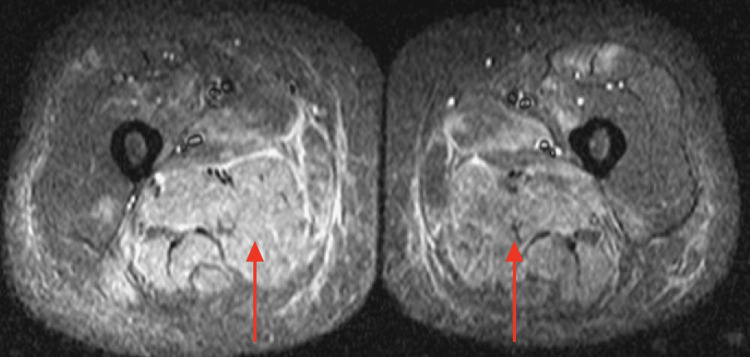
MRI of Bilateral Legs Revealing Edema Within the Muscles Increased signaling in the bilateral adductors, indicated with red arrows, as well as the right semimembranosus and semitendinosus, indicating edema within the muscles.

Electromyography (EMG) testing was conducted, which revealed spontaneous activity within 11 out of 13 muscles tested. Magnetic resonance imaging (MRI) was performed and identified edema within multiple muscles throughout the axial and appendicular skeleton, including the deltoids, latissimus dorsi, iliacus, iliopsoas, gluteus minimus and medius, and adductors (Figures 1-2).

**Figure 3:**
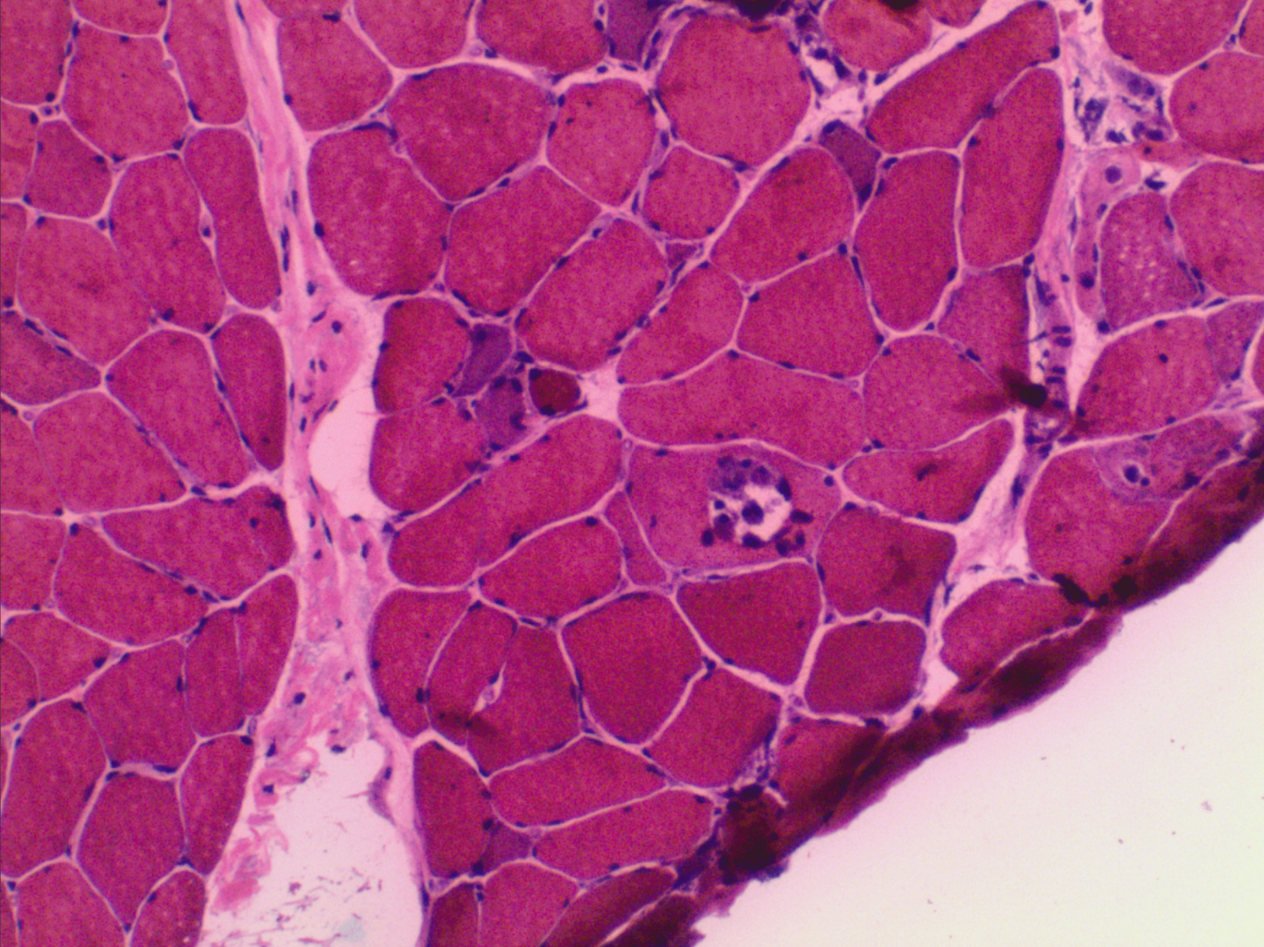
Skeletal Muscle Biopsy Showing Necrotizing Myopathy Frozen hematoxylin and eosin stain (20× magnification) of skeletal muscle biopsy shows a necrotizing myopathy with a myophagocytic fiber and regenerating myofibers.

The patient underwent a muscle biopsy, which showed necrotizing myopathy with myofiber necrosis, regeneration, and myophagocytosis (Figure 3).

Together, the clinical picture was consistent with a diagnosis of SINAM. The patient was started on high-dose prednisone, however, the patient continued to suffer from myalgias without significant improvement in her weakness. She was readmitted 2 weeks later for initiation of IVIG.

## Discussion

Statins act primarily by inhibiting the enzyme 3-hydroxy-3-methylglutaryl-CoA reductase, which is the rate-limiting enzyme for cholesterol synthesis. They also induce the expression of low-density lipoproteins (LDL) receptors on the liver, thus increasing the catabolism of these lipoproteins. Together, these effects lower the overall concentration of cholesterol [2]. Unfortunately, muscle toxicity is a well-known adverse side effect of statins. Muscle toxicities seen with statin use range from mild myalgias, rhabdomyolysis, or, as described in our patient, immune-mediated necrotizing myopathy. Unfortunately, the mechanism behind the development of muscle toxicity is not well understood. For myalgias, some studies propose that statins decrease the levels of coenzyme Q10 in the serum, which leads to impaired mitochondrial function in the muscle cells [3]. A hypothesis regarding the more severe necrotizing myopathy suggests that HMG-CoA reductase expressed in muscle cells is upregulated by statins and contributes to the production of the anti-HMGCR antibodies [4].

Patients diagnosed with immune-mediated necrotizing myopathy will demonstrate progressive muscle weakness, elevated CK levels, and abnormal findings on EMG and muscle biopsy [5]. Muscle weakness is often described by patients as an acute to subacute onset and occurs in a symmetrical proximal to distal distribution. In severe cases of SINAM, muscle weakness may be so severe that it results in dysphagia requiring nasogastric feeding or dyspnea leading to respiratory failure requiring intubation [5]. Creatinine kinase levels are also severely elevated, 10-100 times the upper limit of normal. Interestingly, CK levels often correlate with the level of autoantibodies [6]. EMG testing will reveal spontaneous activity with fibrillation potentials and short-duration motor unit potentials that are characteristically seen in myopathies [5]. Finally, muscle biopsy provides pathologic evidence of muscle fiber necrosis with regeneration and mild inflammation, with mainly macrophages present [6].

While statin-induced myalgias often resolve after discontinuation of the statin, SINAM will have persistent symptoms and require treatment with immunosuppressants [3]. Initial treatment of choice involves daily high-dose corticosteroids (1 mg per kilogram) with or without the addition of IVIG [6]. When first-line treatments are not sufficient, the addition of azathioprine, methotrexate, or mycophenolate mofetil is considered. In those patients with severe weakness or failure to respond to treatment, rituximab can be added after 8-12 weeks [6]. Response to treatment is monitored by symptom reduction as well as CK levels returning to normal levels. Additionally, patients remain on maintenance treatment until they regain full strength. In our patient’s case, her symptoms persisted despite high-dose steroids, so she was readmitted for initiation of IVIG. She has since reported improvement in her weakness, and her CK levels continue to decline. She remains on IVIG every four weeks with plans to slowly taper off prednisone. Once being heavily reliant on a walker to ambulate, she now is using a cane at most.

## Conclusions

Statins are a generally safe and well-tolerated medication prescribed to a large majority of our population. However, SINAM, although rare, can occur. Patients will develop myalgias and weakness that do not resolve with cessation of the medication. This diagnosis should be considered with elevated creatinine kinase, the presence of autoantibodies, spontaneous activity on EMG, and biopsy evidence of muscle necrosis. Patients require immediate statin cessation and initiation of immunosuppressive treatment. Ultimately, patients have favorable outcomes and resolution of their symptoms with prompt recognition and appropriate treatment.
